# Wentong decoction cures allergic bronchial asthma by regulating the apoptosis imbalance of EOS

**DOI:** 10.1186/s13020-018-0180-2

**Published:** 2018-04-18

**Authors:** Yue Yan, Hai-Peng Bao, Chun-Lei Li, Qi Shi, Yan-Hua Kong, Ting Yao, You-Lin Li

**Affiliations:** 1 The 2nd Department of Pulmonary Disease in TCM, The Key Unit of SATCM Pneumonopathy Chronic Cough and Dyspnea, Beijing Key Laboratory of Prevention and Treatment of Allergic Diseases With TCM (No. BZ0321), Center of Respiratory Medicine, China-Japan Friendship Hospital, National Clinical Research Center for Respiratory Diseases, Beijing, 100029 China; 2 Beijing University of Chinese Medicine, Beijing, 100029 China

**Keywords:** Apoptosis, Eosinophil, Bronchial asthma, Wentong decoction

## Abstract

**Background:**

Eosinophils (EOS) is one of the most important cells involved in the pathogenesis of chronic airway inflammation in asthma, and its apoptosis is part of the mechanisms of asthma. Therefore, this study aimed to observe the effect of Chinese medicine Wentong decoction (WTD) in EOS apoptosis in asthmatic rats. This work also explored the mechanism of WTD regulation in EOS apoptosis and provided a new target for clinical treatment of asthma.

**Methods:**

Asthmatic rats induced by ovalbumin were treated with WTD. Lung function of rats in each group was detected, and lung tissue pathology, EOS counts in blood and bronchoalveolar lavage fluid were observed. The degree of the EOS apoptosis in rats was detected. The expression content of interleukin (IL)-5, IL-10, chemokine (C–C motif) ligand 5 (CCL5), granulocyte–macrophage colony-stimulating factor (GM-CSF), transforming growth factor beta 1 (TGF-β1), interferon (IFN)-γ, and other cytokines in rat serum and the genes of Eotaxin mRNA, Fas mRNA, FasL mRNA, Fas/FasL and Bcl-2 mRNA in the lung tissues were determined.

**Results:**

WTD can reduced airway resistance in rat models and improved airway compliance. The pathological changes of lung tissue in WTD group were significantly alleviated, at the same time, WTD could reduce the EOS count in the blood and BALF smears of the asthmatic model rats. Compared with the model group, the apoptosis degree of EOS significantly increased in rats in the WTD group. The expression of IL-5, CCL5, and GM-CSF in the serum and the expression of Eotaxin mRNA, Bcl-2 mRNA in the lung tissues in rats in the WTD group rats decreased. Moreover, the expression of IL-10, TGF-β1, and IFN-γ in the serum and the expression of Fas mRNA, FasL mRNA in the lung tissues in rats in the WTD group rats increased compared with that in rats in the model group.

**Conclusions:**

Wentong decoction may accelerate EOS apoptosis, reduce asthma inflammation, and alleviate the disease through regulating and controlling the factors related to the anti-apoptosis and pro-apoptosis.

**Electronic supplementary material:**

The online version of this article (10.1186/s13020-018-0180-2) contains supplementary material, which is available to authorized users.

## Background

Bronchial asthma (BA) is a chronic inflammatory disease of the airway associated with multiple cells (such as eosinophils, mast cells, T-lymphocytes, neutrophils, and airway epithelial cells) and the cellular elements [1]. This inflammation causes the susceptible person to exhibit high-airway reactivity to each kind of motivating factor causing airway constriction, wherein eosinophil (EOS) infiltration of airway leads to airway inflammation and airway pathology change is an important sign of clinical changes of asthma disease [2, 3]. Asthma is currently incurable, but it can be controlled by appropriate medications, self-management education, and avoidance of exposure to environmental allergens and irritants [4]. Rapid development of global industrialization, environmental pollution, and the change of climate and ecological environments cause rapid increase in global respiratory diseases. Asthma prevalence rate is significantly different in different countries and regions, and the asthma prevalence rate in children is 3.3–29% [5, 6]. The prevalence rate of adult asthma is 1.2–25.5% [7].

Many inflammatory cell infiltrations exist in the bronchial lung tissues of asthmatic patients, and EOS with abnormally long life is the main inflammatory component of allergic reaction in asthmatic patients [8]. BA animal model [9] showed that EOS infiltration is specifically correlated to the increase of airway reactivity and continues to accumulate and activate in the lungs. EOS infiltration also plays an important role in the formation of airway inflammation and the asthma onset [10]. EOS apoptosis plays a key role in the elimination of BA airway inflammation [11], which is associated with the increase of apoptosis, the improvement of BA symptom, and the decrease of inflammation.

Apoptosis, also known as programmed cell death (PCD), is an energy-consuming process, wherein cells obtain a certain signal or undergo some stimulations and an active apoptotic-related gene interacting to produce cells dies out. Simon et al. [12] suggested that EOS may be regulated by both apoptotic and anti-apoptotic signals, and the imbalance of EOS’ anti-apoptosis and pro-apoptosis mechanism may be the root cause of the delay of EOS apoptosis. Studies have shown that the expression of cytokines, growth factors, and cell-surface molecules and their ligands is directly related to EOS apoptosis. In addition, various factors can achieve the effect of resisting apoptosis and promoting apoptosis by different mechanisms, thereby constituting a complex regulatory network, which plays an important role in the occurrence, development, and outcome of asthma. Interleukin (IL)-5, granulocyte–macrophage colony-stimulating factor (GM-CSF), Eotaxin, Bcl-2 and chemokine (C–C motif) ligand 5 (CCL5) are the main factors that maintain the EOS survival and inhibit their apoptosis [13–15]. IL-10, transforming growth factor beta 1 (TGF-β1), interferon (IFN)-γ, and Fas antigens, Fas ligand are involved in the regulation of the EOS apoptosis. These cytokines can also promote EOS apoptosis [16–18].

This experiment reveals the biological effects of WTD on EOS apoptosis in asthmatic rat models and determines the molecular mechanism of WTD in EOS apoptosis by detecting IL-5, IL-10, GM-CSF, CCL5, TGF-β, IFN-γ, Eotaxin mRNA, Bcl-2 mRNA, Fas mRNA, FasL mRNA and other related factors.

## Methods

The minimum standards of reporting checklist (Additional file 1) contains details of the experimental design, and statistics, and resources used in this study.

### Herbal medicine and preparation of Wentong decoction

Twelve herbs, namely, *Astragalus membranaceus*, *cassia twig*, *Rhizoma zingiberis*, *bighead atractylodes rhizome*, *Fructus Corni*, *Rhizoma anemarrhenae*, *Aster tataricus* *Linn*, *Aceranthus sagittatus* *S*. *et* *Z*., *Inula britannica chinensis*, *Magnolia officinalis*, *Schisandra chinensis*, *liquorice*, for Wentong decoction were purchased from Tong Ren Tang (Tong Ren Pharmaceutical Co., Ltd., Beijing, China). Testing shows that the herbal medicines reached the Pharmacopoeia standard (version 2010). The extraction of active constituents from Wentong decoction was performed using water boiling method, and all herbs were made into medicinal extract according to the standard of the School of Chinese Materia Medica, Beijing University of Chinese Medicine.

### Animal handling

Healthy male 4-week-old SD rats were purchased from Beijing Huafukang Bio-Tech Co., Ltd., Beijing, China (License No.: SCXK (Beijing) 2009-0007) and provided adaptive feeding, ambient temperature of 24 ± 2 °C, air humidity of 45–65%, separate cages for natural and artificial light, free diet, and drinking water. The experiment was started 1 week later. All experimental procedures were carried out in accordance with internationally recognized guidelines for the use and care of American Laboratory Animals (NIH Publication No. 85–23, revised in 1985).

Rats were randomly divided into four groups (10 per group) as follows: the normal control group (N), asthmatic rat model group (M), dexamethasone-positive control group (D), and Wentong decoction treatment group (W).

### Establishment of asthmatic rat model

All rats except the normal control group were provided 0.2 mL 10% ovalbumin (OVA)/Al(OH)_3_-mixed liquid (OVA, a16951, Alfa Aesar, Ward Hill, MS, USA; Al(OH)_3_, a4682, Sigma Aldrich, St Louis, MO, USA) at the 1st and 8th days with five-point subcutaneous injection (bipedal, double groin, and peritoneal). At the same time, 0.0023‰ pertussis toxoid (p7208, Sigma-Aldrich) intraperitoneal injection was provided. After the initial sensitization, daily application of 1% OVA saline-atomization inhalation simulated for 1 h was performed using an ultrasonic atomizer (402ai; Yuyue Medical Equipment Co., Ltd., Jiangsu, China), and allergic asthmatic rat model was replicated for 9th–15th days. In the 16th–29th days, the dexamethasone control group and Wentong decoction treatment group were provided 0.5 mg/kg of dexamethasone (Sigma-Aldrich) and 1.34 g/kg of Wentong decoction, respectively, with daily lavage once each day. The normal control group and model group rats were provided equal saline (0.3 mL) for lavage. After 24 h of lavage, the lung tissue, BALF, abdominal aorta blood, and caudal venous blood were collected in rats.

### Lung function test

After intraperitoneal injection of 4% pentobarbital sodium anesthesia in rats (2 mL/kg), rats were positioned in supine position in the enclosure, and 2 mm plastic tube was inserted into the trachea. The plastic tube was connected to a AniRes2005 small animal lung function analyzer (Beijing Bellambo High-tech Co., Ltd., Beijing, China). After 5 min, the inspiratory resistance, expiratory resistance, and dynamic compliance of the airway were observed.

### Lung histopathology and detection of the EOS apoptosis

For lung histopathology and detection, the right lung middle lobe was used, and 4% of paraformaldehyde solution was added fixed for 24 h and paraffin-embedded at 4 µm thick continuous slices for routine hematoxylin and eosin (H&E) staining. In situ end labeling was performed using terminal deoxynucleotidyl transferase dUTP nick end labeling (TUNEL) alkaline phosphatase to detect the apoptotic cells, following the specific operation in strict accordance with the TUNEL reagent box manual (11684817910, Roche, United States). By combining the same H&E staining slices, one observer randomly selected five high-power fields in each slice to determine the index of EOS apoptosis and calculate its average as the representative value of the slice. The observation and analysis of the slices were performed under a light microscope (Olympus Corp., Tokyo, Japan).

### EOS count in blood and BALF smears and flow cytometry technique for the detection of EOS apoptosis in blood

We took 20 µL of the rat tail vein blood, and 0.38 mL of the EOS count liquid was added at static pressure for 30 min. The EOS was counted on the cell count plate, and the procedure was repeated thrice to obtain the mean value.

The BALF was centrifuged (3500 r/min, 15 min), the centrifuged pellets were shaken with 8 mL PBS, and the supernatant was discarded by centrifugation and repeated three times. Take 0.1 mL liquid for smear, H&E staining, 200 cells were counted under a 400-fold light microscope, eosinophils were counted, repeated 3 times, and the average value was taken.

Up to 3 mL abdominal aorta blood was aseptically extracted from rats, and sodium citrate (130 mmol/L, ph 7.4) and 5% fetal bovine serum/Hanks liquid dilution were added for anti-coagulation. In a 15-mL centrifuge tube, we added 1 mL of Percoll liquid with a density of 1.100 and 1.085 g/mL successively. Finally, the diluted blood was added slowly to the cell suspension and horizontally centrifuged at 960×*g* for 15 min (20 °C). After centrifuging, we recycled the eosinophilic cell layer of Percoll liquid interfaces with different densities. D-Hanks liquid was used for centrifugal washing at 1500 rpm for 10 min twice. Then, using D-Hanks liquid of 1 mL heavy suspension, we calculated the absolute numbers of EOS under a microscope. We adjusted the cell count to approximately 2 × 10^6^/mL and took 1 mL for flow detection.

### Enzyme-linked immunosorbent assay (ELISA)

We took 2 mL of the rat’s blood at room temperature, centrifuged at 3000 r/min for 15 min at static pressure for 2 h, and then stored at − 80 °C after collecting the supernatant liquid. We performed the procedure in strict accordance with the ELISA Kit manual. We used the double antibody sandwich-ELISA assay test, to determine the IL-5, IL-10, GM-CSF, CCL5, TGF-β, and IFN-γ (Abcam, Cambridge, UK) in the serum.

### RNA extraction and real-time polymerase chain reaction (PCR)

We evaluated the effect of Wentong decoction on the expression of Eotaxin mRNA and Fas mRNA in the lung tissues of asthmatic rats by using real-time fluorescent quantitative PCR. We determined the primer sequences using glyceraldehyde 3-phosphate dehydrogenase (GAPDH) and performed primer design (Table 1). We used Trizol total RNA extraction kit (DP405-02, Tiangen Biochemical Technology, Beijing Co., Ltd., Beijing, China) to extract total RNA from the 25 mg lung tissue according to the manufacturer’s instructions for inverse transcription of RNA samples to obtain the corresponding cDNA. After pretreatment of the upper machine mixture, we operated the real-time PCR device at 95 °C, 30 s, and 40 PCR loops (collected the fluorescence at 95 °C for 5 s and 60 °C for 40 s). The target and internal control genes of each sample underwent real-time PCR, and the data were analyzed using 2^−△△CT^ method.

**Tabel 1:** Sense and antisense primer sequences of Eotaxin, Bcl-2, Fas, FasL and GAPDH

Equipment name	Primer sequence (5′–3′)	Product size (bp)
GAPDH upstream primer	CCTTCCGTGTTCCTACCCC	131
GAPDH downstream primer	GCCCAGGATGCCCTTTAGTG	
Eotaxin upstream primer	GCTACAAAAGAATCACCAACAACAG	95
Eotaxin downstream primer	CTTTTTCTTGGGGTCAGCACAG	
Bcl-2 upstream primer	GGGCTACGAGTGGGATACTGGAG	101
Bcl-2 downstream primer	CGGGCGTTCGGTTGCTCT	
Fas upstream primer	ATCAATAATCATGGCTGTGT	116
Fas downstream primer	TATTTGAGTGTATCCCTGCT	
FasL upstream primer	GGTGCTGGTGGCTCTGGTT	142
FasL downstream primer	TGTGCTGGGGTTGGCTATTT	

### Data analysis

All data were analyzed using SPSS software (version 17.0, SPSS, Inc., Chicago, IL, USA). The result of each group is shown in mean ± standard deviation. *P* < 0.05 indicates statistically significant difference. Single factor variance analysis and Newman–Keuls test were used for group analysis. If the data do not conform to normal distribution, the nonparametric Kruskal–Wallis test was used for comparison.

## Results

### Lung function

**Fig. 1:**
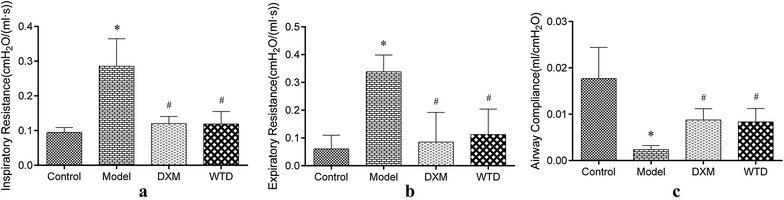
Changes of lung function in rats in each group. **a** Changes in inspiratory resistance of the airway in rats. Inspiratory resistance of asthmatic rat model was significantly higher than that of the normal control group, and inspiratory resistance decreased after DXM and WTD treatments. **b** Changes in airway expiratory resistance in rats. The expiratory resistance of the asthmatic rat model was significantly higher than that in the normal control group, and the expiratory resistance decreased after DXM and WTD treatments. **c** Changes in the dynamic compliance of the airway in rats. Airway compliance in asthmatic rat model was significantly lower than that in the normal control group, and the expiratory resistance increased after DXM and WTD treatments (*p < 0.05, compared with the normal group; ^*#*^*p *< 0.05, compared with the model group; *n *= 8)

Lung function examination plays an important role in evaluating the asthma severity, prognosis, and curative effect of drugs. To evaluate the effect of Wentong decoction in improving lung function in asthmatic rats, we tested the inspiratory resistance, expiratory resistance, and dynamic compliance of the airway in all groups of rats (Fig. 1). The results showed that compared with the normal group, the inspiratory resistance and expiratory resistance of rats in the model group increased significantly. Airway compliance significantly reduced, and the difference was statistically significant (*p *< 0.05). After drug intervention, dexamethasone (DXM) and WTD significantly reduced inspiratory resistance and expiratory resistance in rat models and improved airway compliance (*p *< 0.05). This finding suggested that WTD exhibited positive effect on the improvement of lung function of rats with OVA-sensitized asthma.

### Pathology

**Fig. 2:**
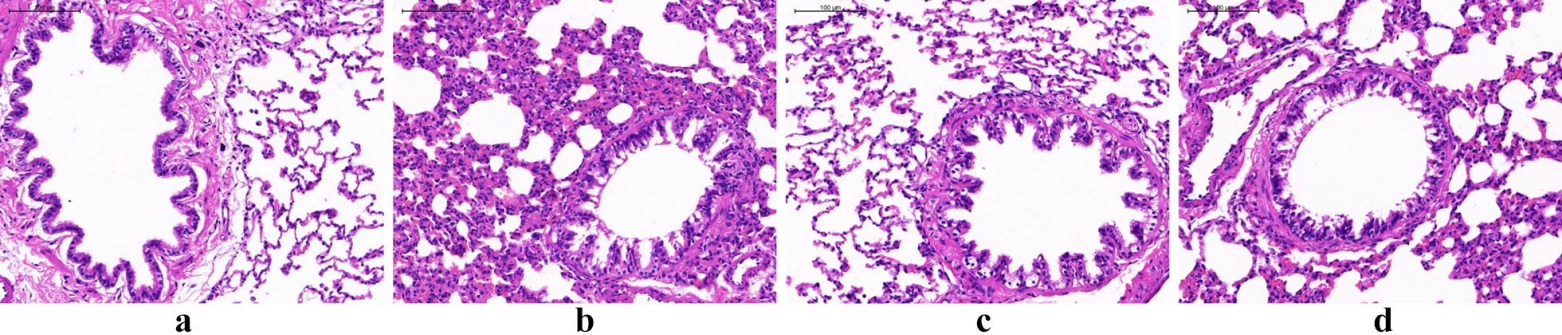
Pathological changes of lung tissue in rats observed by a light microscope: **a** normal control group; **b** model group; **c** DXM group; **d** WTD group

The pathological changes of lung tissue in rats were evaluated using H&E staining in the paraffin section. In the model group, we can observe the irregular dark red hyperemia in the lung tissue, bronchial and vascular smooth muscle hyperplasia, airway and blood vessels with a large number of red-stained eosinophilic granulocyte and inflammatory cell infiltration taking purple-blue cluster-like lymphocytes as principal cells, lumen stenosis, tube wall thickening, arrangement disorder of bronchial mucosa epithelial cell, flakiness falling off, obvious goblet cell hyperplasia, and other typical pathological manifestations of BA. After the intervention of DXM and WTD, the pathological changes of the two groups significantly reduced, and the decrease in pathological changes in the DXM group was significant (Fig. 2). The results showed that WTD and DXM showed similar antiasthma effects in pathological changes of allergic asthma rats.

### EOS count in blood and BALF

**Fig. 3:**
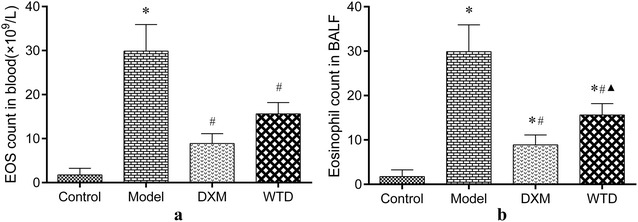
Analysis results of EOS count showing that WTD can reduce the inflammatory level of asthma. **a** EOS count in blood (1 × 10^9^/L; *p < 0.05, compared with the normal control group; ^*#*^*p *< 0.05, compared with the model group; *n *= 8); **b** EOS count in BALF smears (*p < 0.05, compared with the normal control group; ^*#*^*p *< 0.05, compared with the model group; ^▲^*p *< 0.05, compared with the DXM group; *n *= 8)

**Fig. 4:**
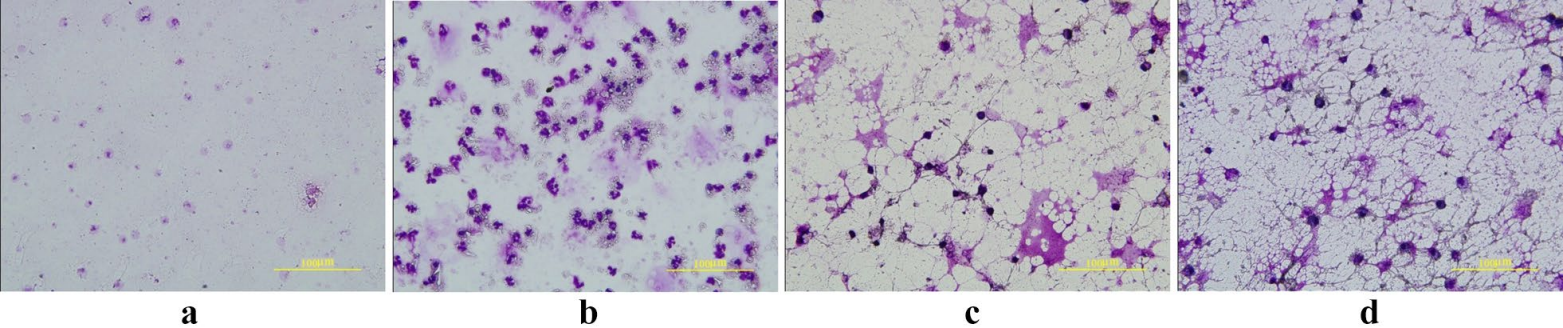
Observation of EOS in BALF under the microscope. Eosinophils are round and 13–15 μm in diameter. The cytoplasm is filled with coarse, neat, even, and tightly arranged brick red or bright red eosinophilic particles. The nucleus is a characteristic lobular nucleus, usually 2–3 leaves, spectacle-shaped, dark purple. Eosinophils are easily broken and particles can be dispersed around the cells. Under the microscope, the expression of eosinophils from high to low was as follows: **b** asthma model group, **d** WTD group, **c** DXM group and **a** normal control group

The inflammatory level of asthma was evaluated by calculating the EOS count of rat-tail-vein blood (Fig. 3a). Observation of BALF smears after HE staining (Fig. 4), counting the number of EOS per 200 cells (Fig. 3b). WTD and DXM can significantly reduce the level of EOS in the blood and BALF of OVA-sensitized-asthmatic rats. The results suggested that WTD may be able to reduce the inflammatory state of asthmatic rats to achieve the effect of antiasthma.

### Detection of EOS apoptosis

**Fig. 5:**
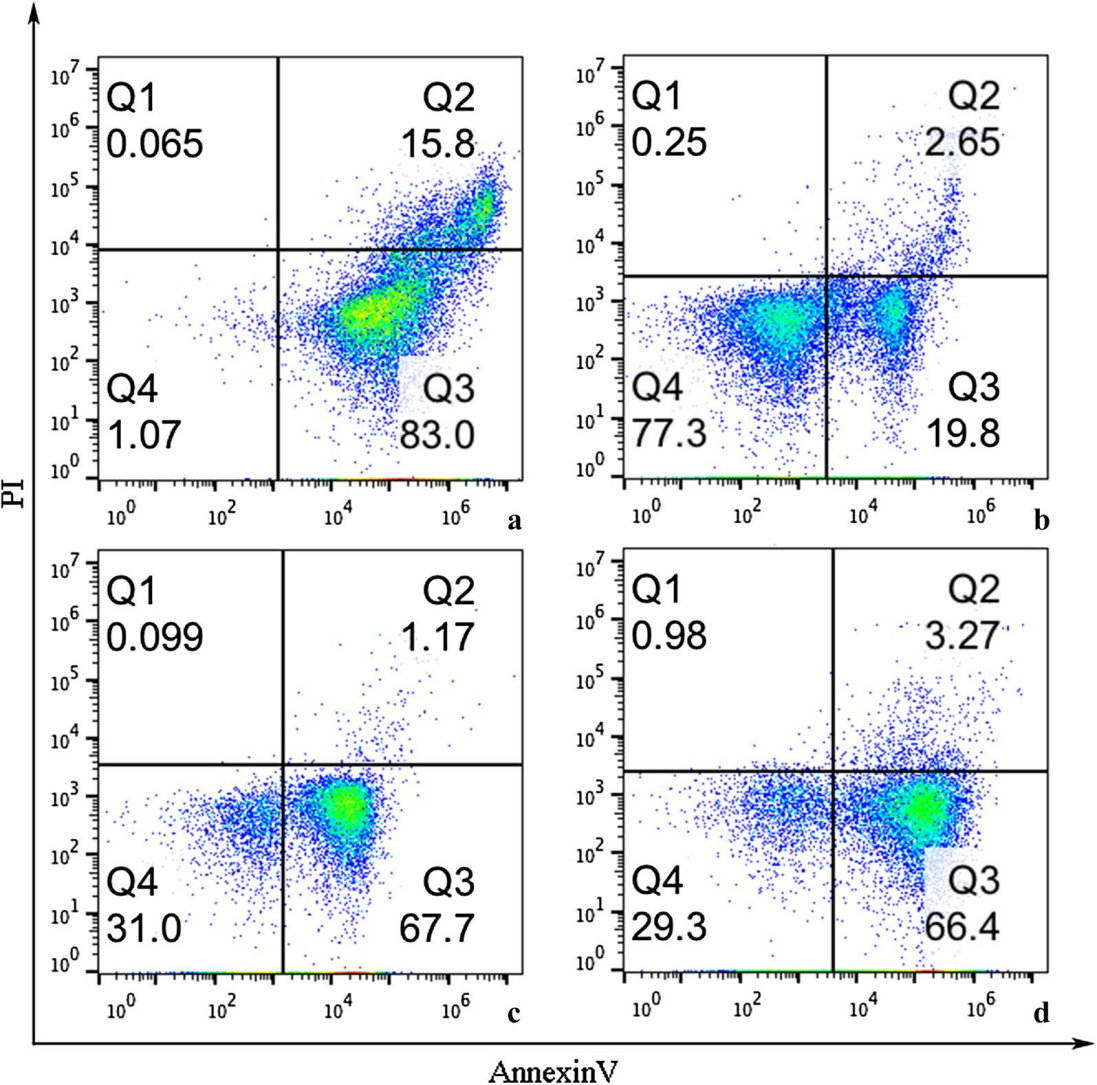
EOS apoptosis in arterial blood of rats: Q3. Quadrant representing the EOS apoptosis; **a** normal control group; **b** model group; **c** DXM group; **d** WTD group

**Fig. 6:**
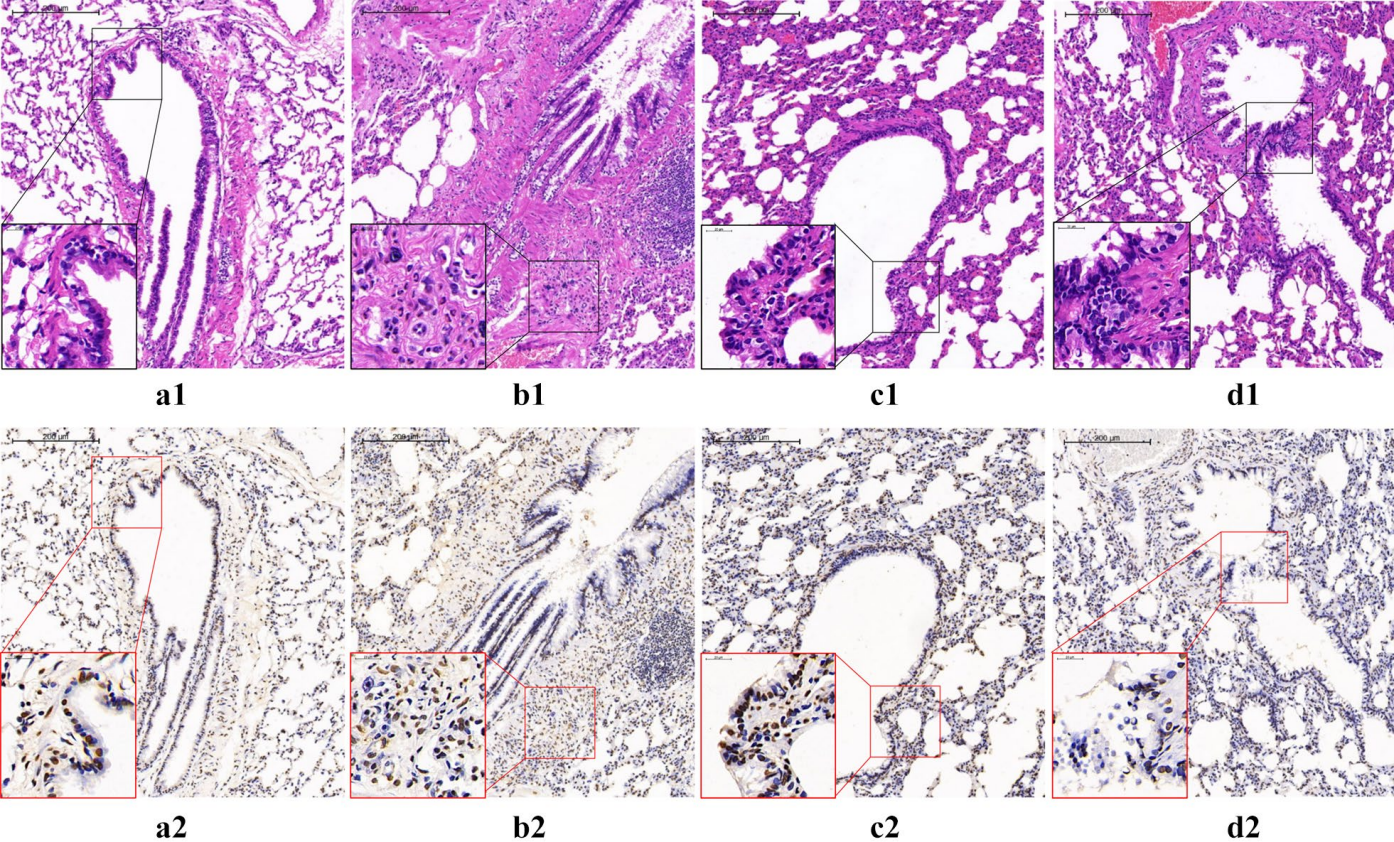
EOS apoptosis in lung tissue of rats: (**a1**, **2**) Normal control group: The structure of bronchopulmonary tissue is normal, the lumen is smooth and the alveolar septum is intact. The eosinophil infiltration can be seen occasionally, and only a small amount of eosinophil apoptosis was found. (**b1**, **2**) Model group: The bronchial lumen is stenotic, the epithelial cells proliferate and fall off, the tracheal smooth muscle and the alveolar septum thickens, and the structure is disordered. A large number of inflammatory cells, mainly eosinophils, infiltrating and apoptosis of eosinophils can be seen. (**c1**, **2**) DXM group: Compared with the model group, the inflammatory cell infiltration of bronchopulmonary tissue in rats was alleviated, the pathological damage was significantly reduced, the apoptosis of EOS and the apoptotic index increased. (**d1**, **2**) WTD group. Showed similar effects to DXM group in reducing pathological injury and increasing EOS apoptosis index in rats

To evaluate the EOS apoptosis in asthmatic rats and the effect of WTD on the degree of EOS apoptosis, we adopted flow cytometry and TUNEL method, respectively, to determine the EOS apoptosis in the arterial blood and lung tissues of rats (Figs. 5, 6). The results showed that the degree of EOS apoptosis in arterial blood and lung tissues of rats was obviously consistent. Compared with the normal control group, the percentage of EOS apoptosis in asthmatic rat model decreased significantly, and the difference was statistically significant (*p *< 0.05). Compared with the model group, the WTD group could significantly increase the percentage of serum EOS in rats, and the difference was statistically significant (*p *< 0.05).

### Expression of cytokines in the serum

**Fig. 7:**
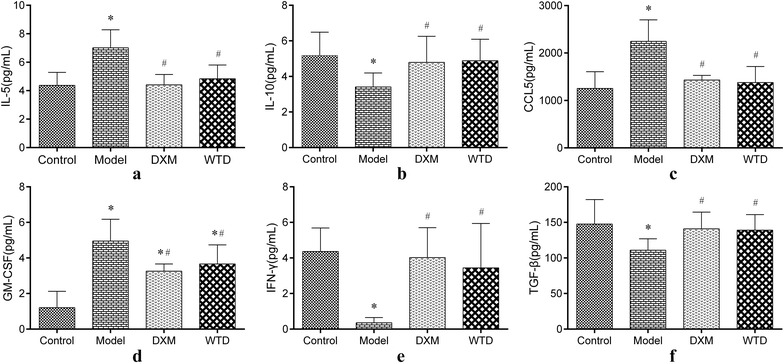
Changes of cytokines in rat serum: **a**, **c**, and **d** Expression of IL-5, CCL5, and GM-CSF. The expression of IL-5, CCL5, and GM-CSF in asthmatic rat model was significantly higher than that in the normal control group, and the expression of IL-5, CCL5 and GM-CSF after DXM and WTD treatments significantly reduced. **b**, **e**, and **f** Expression of IL-10, IFN-γ, and TGF-β. The expression of IL-10, IFN-γ, and TGF-β in asthmatic rat model was significantly lower than that in the normal control group, and the expression of IL-10 and IFN-γ, and TGF-β significantly increased after DXM and WTD treatments. (*p < 0.05, compared with the normal group; ^*#*^*p *< 0.05, compared with the model group; *n *= 8)

Several kinds of cytokines will exhibit a certain effect on EOS apoptosis. To further clarify whether the WTD can adjust the apoptosis state of EOS to achieve the purpose of alleviating asthma, we use ELISA method to detect IL-5, IL-10, CSF, CCL5, TGF-β, and IFN-γ in rat serum (Fig. 7). The results show that WTD and DXM can significantly reduce the expression of IL-5, GM-CSF, and CCL5 and significantly enhance the expression of IL-10, TGF-β, and IFN-γ in asthmatic rats.

### Expression of Eotaxin, Bcl-2, Fas and FasL mRNA in the lung tissues

**Fig. 8:**
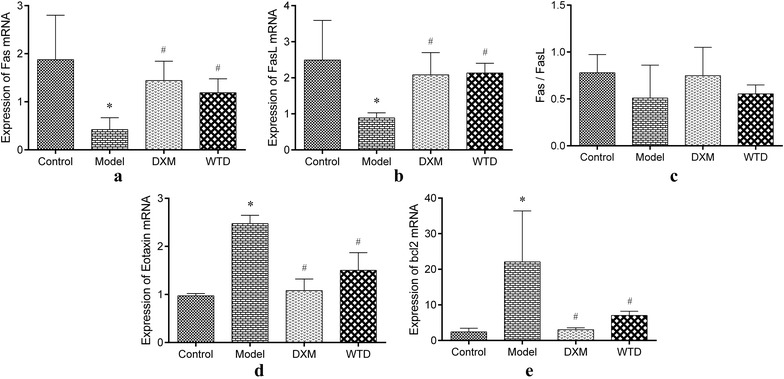
Gene expression of Eotaxin, Bcl-2, Fas and FasL in lung tissues. **a**, **b** Compared with the normal control group, the expression of Fas and FasL mRNA in lung tissues of the asthmatic model group significantly lower, the Fas and FasL mRNA increased after DXM and WTD treatment; **c** the expression of Fas and FasL was consistent, and the ratio of Fas/FasL showed no significant difference among groups. **d**, **e** Both the expression of Eotaxin and Bcl-2 mRNA in lung tissues of the asthmatic model group was significantly higher than that of the normal group. The Eotaxin and Bcl-2 mRNA decreased after DXM and WTD treatment (*p < 0.05, compared with the normal group; ^#^*p *< 0.05, compared with the model group; n = 4)

Real-time PCR assay was used to detect the gene expression of Eotaxin, Bcl-2, Fas and FasL in lung tissues of rats. Quantitative results show that the expression of Eotaxin and Bcl-2 mRNA in lung tissue of the asthmatic model group significantly increased, whereas the expression of Fas and FasL mRNA decreased (compared with normal control group). Both DXM and WTD can significantly reduce the expression of Eotaxin and Bcl-2 mRNA in lung tissues of model rats and significantly increase Fas and FasL mRNA content in the lung tissues of rats (Fig. 8).

## Discussion

In this experiment, we successfully established asthmatic rat model by OVA sensitization, weakened pulmonary-function state in the model group, deteriorated histopathological changes, and aggregated and infiltrated the EOS. These undertakings can prove the success of our asthmatic rat model. After the intervention of WTD and DXM, the condition of rats improved obviously, indicating that WTD can achieve the asthma-like effect of DXM. In addition, we also found that WTD showed some anti-asthmatic effects in a dose-dependent manner. The WTD middle-dose group had more pronounced anti-asthma effects than the low-dose group, but the high-dose group and middle-dose groups had similar efficacy, Medium dose group is human equivalent group.

EOS infiltration is one of the important inflammatory mechanisms of asthma, and the activation and long-term survival of EOS are concentrated in the bronchial bronchi of allergic asthma [19], which induces a series of symptoms. However, the delay of EOS apoptosis maintained and aggravated the inflammatory state, which increased the difficulty of treatment. After bone marrow maturation, the EOS circulates briefly (~ 3 days) causing the PCD (apoptosis) in the peripheral blood [8]. Meanwhile, the apoptosis and necrosis are different, apoptosis indicating positive biological significance necessary for maintaining organism stability. The results of this experiment show that WTD can promote the EOS apoptosis in the lung tissue and blood and alleviate the inflammatory state of asthma.

IL-5 is a glycoprotein composed of 115 amino acids and mainly produced by T-helper cells and plays a biological effect through the binding of target cell surface of the specific receptor. IL-5 exhibits the role of promoting the proliferation, activation, and release of inflammatory mediators of EOS. IL-5 is also the most important eosinophil promoter. Suzuki [20] studies have confirmed that IL-5 inhibits EOS apoptosis. The high-affinity receptors on the EOS membrane combine with IL-5 to transmit the anti-apoptotic information into the cells, inhibit the apoptosis, and maintain the cell survival [13, 21].

CCL5 displays chemotaxis to EOS, which can show activation of EOS in vitro and increase EOS to express intercellular adhesion molecules. The study found that the CCL5 level in bronchoalveolar lavage fluid is significantly high in asthmatic patients [22], and the polymorphism of CCL5 gene is a risk factor for asthma by using meta-analysis of Huang et al. [23].

Eotaxin is one of the members of the CCL family. In normal respiratory tract, Eotaxin is mainly produced by epithelial cells. However, the exudation macrophages and EOS after antigen sensitization to some extent are the main sources of Eotaxin. This condition increases the Eotaxin formation [24]. Dexamethasone inhibits the production of Eotaxin mRNA in EOS, and IL-5 can induce Eotaxin expression in EOS [25]. Eotaxin increased synthesis after antigen stimulation, promoting the selective recruitment of EOS in local tissues. Once a large amount of EOS accumulated at the antigen-stimulating site, IL-5, together with other factors, causes the EOS to prolong survival and release a range of protein particles, leading to asthma attacks. Bcl-2 gene is a proto-oncogene that inhibits apoptosis. It not only plays an important role in the molecular regulation of apoptosis, but also has a close relationship with the occurrence and development of asthma. The high expression of Bcl-2 gene inhibits the apoptosis of eosinophils, causing a large number of eosinophils to infiltrate into the peripheral blood, lung tissue, and BALF. Eosinophils release inflammatory mediators and lead to asthma attacks.

GM-CSF is a cytokine closely related to asthma pathogenesis [26]. GM-CSF is mainly produced from T cells and macrophages. In asthma pathogenesis, GM-CSF can induce the growth of T cell precursors, cause EOS chemotaxis and activation, and promote EOS collection in the airway, causing airway-epithelial injury, inflammatory cell infiltration, and high-airway reactivity [27, 28].

In this study, we found that the content of serum IL-5, CCL5, GM-CSF, Eotaxin mRNA and Bcl-2 mRNA in asthmatic rat model was significantly higher than that in normal control group and negatively correlated with EOS apoptosis. WTD and DXM can effectively reduce the expression of the three cytokines in the serum of model rats. The effect of DXM on reducing CCL5 content was better than that of TCM Wentong decoction. However, no significant difference was observed between the two groups in IL-5, GM-CSF, and Eotaxin mRNA (*p *> 0.05).

IL-10 is an immunosuppressive agent with a multidirectional biological activity secreted by Th2 cells. IL-10 exhibits a direct inhibition effect on airway inflammatory cell activation, releases inflammatory factor in asthmatic patients [29, 30], can inhibit the IgE production, promotes IgG4 synthesis, and plays an important role in pathophysiology of allergic asthma [31]. Study found that IL-10 can inhibit the ability of Th2 cells to produce IL-5, and its inhibitory effect is concentration-dependent [32].

TGF-β induces EOS apoptosis and inhibits the IL-5, GM-CSF, and other cytokine-induced EOS to prolong the life cycle [33]. In addition to inhibiting cytokine synthesis and EOS survival, TGF-β also prevented EOS from releasing peroxidase and decreased the expression of EOS cell lines (Eol-1) ${\text{CD}}_{ 2 3}^{ + }$, indicating that TGF-β shows an inhibiting effect on growing EOS, differentiation, function, and all aspects of survival [34]. IFN-γ mainly originates from Th1 cells. In vivo experiments showed that IFN-γ can inhibit the infiltration of eosinophilic cells caused by allergens in the lungs [35], and inhibit the synthesis of Th2 cytokine IL-5, and its mechanism may be that IFN-γ inhibits the EOS collection by blocking the IL-5 synthesis by inhibiting GATA3 expression [36].

Fas gene is encoded as one of the members of the TNF receptor family, and its activation can induce apoptosis. Study found that interferon combined with tumor necrosis factor promoted the Fas expression on the surface of EOS and enhanced the FasL-mediated EOS apoptosis in vitro [37]. The expression of Fas receptor and FasL is relatively high in the cells of the immune system, which mediates apoptosis/proliferation plays an important role in asthma T cells with dysregulation of apoptosis/proliferation, Th1/Th2 imbalance, airway inflammation, airway hyperresponsiveness (AHR) and airway remodeling.

In this study, we found that the content of IL-10, TGF-β1, IFN-γ in the serum, Fas and FasL mRNA in the lung tissues of asthmatic rat model was significantly lower than that of the normal control group, which was positively correlated with the EOS apoptosis. Both WTD and DXM can effectively improve the expression of IL-10, TGF-β1, IFN-γ in the serum, and Fas mRNA in the lung tissues of model rats. This condition inhibits the EOS infiltration and promotes the EOS apoptosis to reduce the inflammatory response of asthma and achieve the goal of treating the disease.

In the complex internal environment, the local EOS apoptosis is not determined by a single factor but is controlled by the complex network system consisting of many cytokines, cell surface molecules, ligands, and chemokines. Maintaining homeostasis between pro-apoptotic factors and anti-apoptotic factors is the key to determine the EOS apoptosis.

## Conclusions

Wentong decoction may promote the EOS apoptosis and reduce airway inflammation by increasing the expression of pro-apoptotic factors of IL-10, TGF-β1, IFN-γ, Fas mRNA and FasL mRNA and decreasing the anti-apoptotic factors of IL-5, GM-CSF, CCL5, Eotaxin mRNA and Bcl-2 mRNA. Thus, asthma prevention is achieved.

## Additional file

**Additional file 1.** The minimum standards of reporting checklist.

## Abbreviations

TCM: traditional Chinese medicine
EOS: eosinophil
WTD: Wentong decoction
IL: interleukin
CCL5: chemokine (C–C motif) ligand 5
TGF-β1: transforming growth factor beta 1
GM-CSF: granulocyte–macrophage colony-stimulating factor
IFN-γ: interferon-γ
BA: bronchial asthma
PCD: programmed cell death
DXM: dexamethasone
